# Observed Face Mask Use at Six Universities — United States, September–November 2020

**DOI:** 10.15585/mmwr.mm7006e1

**Published:** 2021-02-12

**Authors:** Lisa C. Barrios, Margaret A. Riggs, Ridgely Fisk Green, Michaila Czarnik, Randall J. Nett, J. Erin Staples, Michael David Welton, Jessica Legge Muilenburg, Keith J. Zullig, Linda Gibson-Young, Andrea V. Perkins, Cindy Prins, Michael Lauzardo, Jerne Shapiro, George Asimellis, Genesia Kilgore-Bowling, Kenny Ortiz-Jurado, Margaret J. Gutilla

**Affiliations:** ^1^CDC COVID-19 Emergency Response Team; ^2^Carter Consulting, Inc., Atlanta, Georgia; ^3^4ES Corporation, San Antonio, Texas; ^4^University of Georgia, Athens, Georgia; ^5^West Virginia University, Morgantown, West Virginia; ^6^Auburn University, Auburn, Alabama; ^7^University of Florida, Gainesville, Florida; ^8^University of Pikeville, Pikeville, Kentucky; ^9^Colorado State University, Fort Collins, Colorado.

Approximately 41% of adults aged 18–24 years in the United States are enrolled in a college or university (*1*). Wearing a face mask can reduce transmission of SARS-CoV-2, the virus that causes coronavirus disease 2019 (COVID-19) (*2*), and many colleges and universities mandate mask use in public locations and outdoors when within six feet of others. Studies based on self-report have described mask use ranging from 69.1% to 86.1% among adults aged 18–29 years (*3*); however, more objective measures are needed. Direct observation by trained observers is the accepted standard for monitoring behaviors such as hand hygiene (*4*). In this investigation, direct observation was used to estimate the proportion of persons wearing masks and the proportion of persons wearing masks correctly (i.e., covering the nose and mouth and secured under the chin*) on campus and at nearby off-campus locations at six rural and suburban universities with mask mandates in the southern and western United States. Trained student observers recorded mask use for up to 8 weeks from fixed sites on campus and nearby. Among 17,200 observed persons, 85.5% wore masks, with 89.7% of those persons wearing the mask correctly (overall correct mask use: 76.7%). Among persons observed indoors, 91.7% wore masks correctly. The proportion correctly wearing masks indoors varied by mask type, from 96.8% for N95-type masks and 92.2% for cloth masks to 78.9% for bandanas, scarves, and similar face coverings. Observed indoor mask use was high at these six universities with mask mandates. Colleges and universities can use direct observation findings to tailor training and messaging toward increasing correct mask use.

Direct in-person observation is used in health care settings to measure adherence to infection prevention and control recommendations, such as hand hygiene and the correct use of personal protective equipment (*4*). A similar approach was used to directly observe mask use at universities, using a protocol and sampling methodology based on one from Resolve to Save Lives, an initiative promoting the measuring and adoption of face mask use to reduce transmission of COVID-19 (*5*). CDC staff members discussed the direct observation protocol with 12 universities, six of which chose to participate in this investigation. The participating universities included five public universities with student populations ranging from 29,000 to 52,000 and one private university with a student population of 2,300; five universities were in the South U.S. Census region (two in East South Central and three in South Atlantic), and one was in the West. Approximately 10 student observers per university were trained by one CDC staff member who conducted training for all participating universities using a standard protocol.^†^ Universities selected approximately 10 observation locations where mask use was mandated.^§^ Indoor mask use was mandated by all selected universities and their surrounding communities. Outdoor mask use was mandated when other physical distancing measures were difficult to maintain.^¶^ Observation locations could be either indoors or outdoors; however, because determining whether persons observed outdoors should have been wearing a mask was not always possible, the analyses focused on indoor mask use. For up to 8 weeks (range: 2 to 8 weeks across universities), observers tracked mask use on varying days and times from fixed sites on campus (e.g., libraries, classroom buildings, dining facility entrances, student centers, and lobbies of recreation centers and workout facilities) and, at five universities, at nearby off-campus, public locations frequented by students (e.g., grocery stores, pharmacies, and cafes). Observers modeled correct mask wearing, remained inconspicuous, and refrained from interacting with the persons they were observing. Each observer was instructed to record 40 observations at a single location or to observe for 1 hour, whichever came first, for a total of approximately 400 observations per week per university by the 10 observers. Correct mask use was recorded if the mask completely covered the nose and mouth and was secured under the chin. Observers were advised to record only what they could see; for example, if a person’s face could not be observed but mask straps were visible behind the person’s head or ears, mask use was recorded as “unknown.” Observers were asked to remain stationary and record 1) whether a mask was worn, 2) whether the mask was worn correctly, and 3) the type of mask worn (cloth, surgical, gaiter, masks that appeared to be N95 respirators [referred to as N95 type], or other) for every third person passing a prespecified location, such as a building entrance. If foot traffic was too high to observe every third person, observers were asked to select every tenth person for the entire observation period (*5*). Observation times varied during the mornings and afternoons and at night and occurred on weekdays and weekends. Because social groups might exhibit more similar mask use behaviors, only one person from a social group (e.g., an easily identifiable family unit, group of friends, or sports team) was sampled to avoid the effects of clustering. Observers were instructed to observe the first person in the group who corresponded to the third person following the preceding observation and then skip remaining group members and resume counting every third person after the group passed. Observations were restricted to persons who appeared to be aged ≥12 years and were not limited to students. One participating university released weekly media reports highlighting their data from this assessment to encourage mask use in their community. A second university released a single media report after 3 weeks of data collection. The remaining four universities did not publicize this investigation.

Data collection was standardized through common training materials and data collection forms to provide comparable data across the six universities. Data were collected using a paper form and entered into REDCap (version 9.7; Vanderbilt University) electronic data capture and management tools hosted at CDC or collected directly using the REDCap tools. Each week, data for each university were compiled and returned to the university, including the proportion of persons observed wearing masks, the proportion of those persons wearing masks correctly, and the most common type of mask worn. Staff members at universities performed quality control processes weekly and provided updated, corrected data to CDC. All analyses were conducted with SAS (version 9.4; SAS Institute). Frequencies and ranges were calculated for mask use, correct mask use, type of mask worn, and locations observed. Chi-squared tests were used to compare indoor mask use and indoor correct mask use for on-campus and nearby off-campus locations. The Tukey honestly significant difference test was used to compare mask types among the proportion used correctly indoors; p-values <0.05 were considered statistically significant. This activity was reviewed by CDC and was conducted consistent with applicable federal laws and CDC policies.**

A total of 17,200 persons were observed at six universities (ranging from 438 persons observed during 2 weeks of data collection to 8,580 during 8 weeks of data collection) (Table 1). Two thirds (66.6%) of the observations took place indoors, and 69% took place on campus. Most (85.5%) observed persons wore masks, with 89.7% of those wearing them correctly (overall correctly wearing masks: 76.7% [range: 72.2%–93.6%]). Cloth masks were most common (68.3%), followed by surgical masks (25.7%). Less common were gaiters (3.8%) and N95-type masks (1.9%). Other face coverings, such as bandanas and scarves, were rarely observed (0.3%). Overall, mask use was significantly more common indoors (94.0%) than outdoors (67.6%) (p<0.001). Among observations conducted indoors, mask use was more prevalent at on-campus (94.8%) than at nearby off-campus locations (90.6%) (p<0.001), as was correct mask use among those wearing masks (92.1% versus 90.0%, respectively; p = 0.002) (Table 2). Correct mask use indoors differed by mask type, with N95-type masks most likely to be worn correctly indoors (96.8%), followed by cloth masks (92.2%), surgical masks (90.8%), gaiters (86.8%), and other face coverings (78.9%) (Table 3). These mask types accounted for 1.7%, 68.2%, 26.1%, 3.7%, and 1%, respectively, of observed masks worn indoors.

**TABLE 1:** Observed number and percentage of persons wearing face masks on six university campuses* and at nearby off-campus locations,^†^ by selected characteristics — United States, September–November 2020

Characteristic	No. (%) of persons observed						
	Total	University A (observed 8 wks)	University B (observed 7 wks)	University C (observed 6 wks)	University D (observed 5 wks)	University E (observed 2 wks)	University F (observed 2 wks)
**Overall mask use**	**17,200 (100)**	8,580 (49.9)	3,144 (18.3)	2,922 (17.0)	1,460 (8.5)	438 (2.5)	656 (3.8)
Mask worn	**14,704 (85.5)**	7,018 (81.8)	2,637 (83.9)	2,619 (89.6)	1,384 (94.8)	430 (98.2)	616 (93.9)
Mask worn correctly	**13,189 (89.7)**	6,434 (91.7)	2,269 (86.0)	2,320 (88.6)	1,171 (84.6)	410 (95.3)	585 (95.0)
**Type of mask**							
Cloth	**10,042 (68.3)**	5,042 (71.8)	1,645 (62.4)	1,587 (60.6)	1,079 (78.0)	278 (64.7)	411 (66.7)
Surgical	**3,774 (25.7)**	1,592 (22.7)	804 (30.5)	839 (32.0)	236 (17.1)	134 (31.2)	169 (27.4)
Gaiter	**563 (3.8)**	200 (2.8)	154 (5.8)	125 (4.8)	56 (4.0)	5 (1.2)	23 (3.7)
N95 type	**280 (1.9)**	175 (2.5)	29 (1.1)	48 (1.8)	10 (0.7)	10 (2.3)	8 (1.3)
Other	**45 (0.3)**	9 (0.1)	5 (0.2)	20 (0.8)	3 (0.2)	3 (0.7)	5 (0.8)
**Location**							
Indoors	**11,451 (66.6)**	4,686 (54.6)	1744 (55.5)	2,758 (94.4)	1,279 (87.6)	438 (100)	546 (83.2)
Outdoors	**5,546 (32.2)**	3,734 (43.5)	1,400 (44.5)	121 (4.1)	181 (12.4)	—^§^	110 (16.8)
On bus	**203 (1.2)**	160 (1.9)	—	43 (1.5)	—	—	—
**Campus**							
On campus	**11,875 (69.0)**	5,884 (68.6)	2,709 (86.2)	905 (31.0)	1,460 (100)	329 (75.1)	588 (89.6)
Nearby off campus	**5,122 (29.8)**	2,536 (29.6)	435 (13.8)	1,974 (67.6)	—	109 (24.9)	68 (10.4)
On bus	**203 (1.2)**	160 (1.9)	—	43 (1.5)	—	—	—

* Includes five public universities with student populations ranging from 29,000 to 52,000 and one private university with a student population of 2,300; five universities were in the South U.S. Census region (two in East South Central and three in South Atlantic), and one was in the West.

^†^ Data are from five universities. Nearby, indoor and outdoor off-campus locations in the surrounding community that were known to be frequented by students (e.g., grocery stores, pharmacies, and cafes) in counties where mask use was mandated indoors or outdoors if 6 feet of distance could not be maintained.

^§^ Data not collected.

**TABLE 2:** Observed overall number and percentage of persons wearing face masks indoors* and wearing face masks indoors correctly on six university campuses^†^ and at nearby, indoor off-campus locations^§^ — United States, September–November 2020

Characteristic	No. (%) of persons observed		
	Total wearing masks	On campus	Nearby off campus
Mask worn indoors^¶^	**10,760 (94.0)**	8,648 (94.8)	2,112 (90.6)
Mask worn indoors correctly**	**9,862 (91.7)**	7,962 (92.1)	1,900 (90.0)

* Indoor, on-campus locations where mask use was mandated (e.g., libraries, classroom buildings, dining facility entrances, student centers, and lobbies of recreation centers and workout facilities).

^†^ Includes five public universities with student populations ranging from 29,000 to 52,000 and one private university with a student population of 2,300; five universities were in the South U.S. Census region (two in East South Central and three in South Atlantic), and one was in the West.

^§^ Data are from five universities. Nearby, indoor off-campus locations in the surrounding community that were known to be frequented by students (e.g., grocery stores, pharmacies, and cafes) in counties where mask use was mandated indoors or outdoors if 6 feet of distance could not be maintained.

^¶^ p<0.001. Total number observed = 11,451, on-campus indoor observed = 9,119, and nearby off-campus observed = 2,332. The chi-squared test was used to assess the difference between masks worn indoors on campus and at nearby off-campus locations in the surrounding community.

** p = 0.002. Total number observed indoors = 10,758, excluding 693 observations (no mask use or unknown mask use) and missing data for two observations. The chi-squared test was used to assess the difference between correct mask use indoors on campus and at nearby off-campus locations in the surrounding community.

**TABLE 3:** Observed number and percentage of persons wearing face masks indoors correctly among all persons wearing face masks on six university campuses* and at nearby, indoor off-campus locations,^†^ by mask type — United States, September–November 2020

Type of mask^§^	Mask worn indoors	Mask worn indoors correctly
	No.	No. (%)
**Total**	**10,760^¶^**	**9,862 (91.7)**
Cloth	7,334	6,760 (92.2)
Surgical	2,807	2,549 (90.8)
Gaiter	394	342 (86.8)
N95 type	187	181 (96.8)
Other**	38	30 (78.9)

* Includes five public universities with student populations ranging from 29,000 to 52,000 and one private university with a student population of 2,300; five universities were in the South U.S. Census region (two in East South Central and three in South Atlantic), and one was in the West.

^†^ Nearby, indoor off-campus locations in the surrounding community that were known to be frequented by students (e.g., grocery stores, pharmacies, and cafes) in counties where mask use was mandated indoors or outdoors if 6 feet of distance could not be maintained.

^§^ p<0.05. Post hoc comparisons using the Tukey honestly significant difference test indicated differences between mask type and the proportion used correctly indoors. Significant differences were observed between all mask types, except cloth and surgical (p = 0.24), cloth and N95 type (p = 0.18), and gaiter and other (p = 0.32).

^¶^ Total observed indoors = 11,451, excluding 691 observations (no mask use or unknown mask use).

** Other face coverings include bandanas and scarves.

## Discussion

Mask mandates have been shown to decrease SARS-CoV-2 case transmission,^††^ and widespread mask use is a core intervention for curbing the COVID-19 pandemic (*6**,**7*). Direct observation at six universities indicated that mask use was high on campuses in locations where masks were mandated. Mask use was similarly high at nearby, indoor off-campus locations where masks were mandated. Mask use was lower outdoors in areas where use was mandated only when physical distancing could not be maintained. These data provide evidence that adherence to university mask mandates is high (*5*). However, correct mask use varied by mask type.

Universities have several opportunities to enforce policies such as mask mandates. For example, universities could impose sanctions for noncompliance with university policy. Universities also could use multimodal education and messaging to reinforce mask use, as well as messaging specific to mask type and that is focused on correct use. One university found that having students sign a compact agreeing to mask use, physical distancing, and testing might also be effective in promoting these behaviors (*8*).

Observational investigations can provide rapid feedback to universities on the prevalence and type of mask use in their population. Using trained student volunteers, participating universities can quickly organize and collect substantial amounts of data weekly at low to no cost and review the data quickly to assess and report on mask use. Universities and their communities can use these data to tailor and evaluate the effectiveness of messages and education to reinforce and increase mask use and to identify locations with lower adherence for policy enforcement.

The findings in this report are subject to at least three limitations. First, because the period of observation ranged from 2 to 8 weeks among universities, overall percentages are influenced by the universities with more data. However, all six universities are continuing to collect data during the 2021 spring semester. Second, observations were sampled without recording information about the persons observed and were not limited to university students, staff members, or faculty members. Off-campus locations likely included more persons not affiliated with the university, and off-campus percentages should be considered a measure of community mask use. Finally, none of the universities mandated outdoor mask use, unless physical distancing could not be maintained. Observers did not record whether physical distancing was or was not maintained.

Compliance with CDC’s recommended COVID-19 mitigation strategy of mask wearing exceeded 80% at six U.S. universities. Mask use is likely to remain a critical COVID-19 mitigation strategy, and CDC has made the training materials used in this study available for universities that would like to monitor mask use on their campuses. However, in addition to mask mandates, universities have implemented multicomponent strategies that included reduced residential density; surveillance and entry testing; educational campaigns; and other campus and community mitigation strategies. Monitoring mask use, tailoring messages to promote healthy behaviors (e.g., mask use, handwashing, and physical distancing) on and off campus, and developing measures to enforce or ensure compliance with healthy behaviors have the potential to improve implementation and effectiveness of public health strategies to protect persons on campus and in the surrounding communities by preventing the spread of SARS-CoV-2.

**Summary**

**What is already known about this topic?**

Correct use of face masks limits COVID-19 transmission. Many institutions of higher education mandate masks in public indoor locations and outdoors when within six feet of others.

**What is added by this report?**

During September–November 2020, mask use was directly observed at six universities with mask mandates. Among persons observed indoors, 91.7% wore masks correctly, varying by mask type, from 96.8% for N95-type masks and 92.2% for cloth masks to 78.9% for bandanas, scarves, and similar face coverings.

**What are the implications for public health practice?**

Direct observation provides rapid feedback on mask use prevalence. Institutions of higher education can use this feedback to tailor training and messaging for correct mask use.

## Footnotes

*
https://www.cdc.gov/coronavirus/2019-ncov/prevent-getting-sick/how-to-wear-cloth-face-coverings.html

† Protocol, training materials, and data collection form are available. https://www.train.org/cdctrain/course/1094943

§ Locations or situations in which mask use was not mandated (e.g., while eating, exercising in gyms, or in individual rooms) were not included in the observation locations.

¶ Difficulty maintaining physical distancing measures was defined in various ways in university mask policies, including when persons are with others with whom they are not cohabitating, when persons cannot maintain >6 feet of distance from others, and gatherings of ≥10 persons.

** 45 C.F.R. part 46; 21 C.F.R. part 56; 42 U.S.C. Sect. 241(d); 5 U.S.C. Sect. 552a; 44 U.S.C. Sect. 3501 et seq.

††
https://www.medrxiv.org/content/10.1101/2020.10.28.20221705v2
